# Catch-Up Growth Following Fetal Growth Restriction Promotes Rapid Restoration of Fat Mass but Without Metabolic Consequences at One Year of Age

**DOI:** 10.1371/journal.pone.0005343

**Published:** 2009-04-28

**Authors:** Jacques Beltrand, Ramona Nicolescu, Florentia Kaguelidou, Rasa Verkauskiene, Oliver Sibony, Didier Chevenne, Olivier Claris, Claire Lévy-Marchal

**Affiliations:** 1 INSERM, U690, Paris, France; 2 Université Paris 7, Paris, France; 3 CIC 92-02, Hôpital Robert Debré, Paris, France; 4 Service de chirurgie gynécologique-obstétrique, Hôpital Robert Debré, Paris, France; 5 Service de biochimie et hormonologie, Hôpital Robert Debré, Paris, France; 6 Service de médecine néonatale, Hôpital Edouard Herriot, Lyon, France; Institute of Preventive Medicine, Denmark

## Abstract

**Background:**

Fetal growth restriction (FGR) followed by rapid weight gain during early life has been suggested to be the initial sequence promoting central adiposity and insulin resistance. However, the link between fetal and early postnatal growth and the associated anthropometric and metabolic changes have been poorly studied.

**Methodology/Principal Findings:**

Over the first year of post-natal life, changes in body mass index, skinfold thickness and hormonal concentrations were prospectively monitored in 94 infants in whom the fetal growth velocity had previously been measured using a repeated standardized procedure of ultrasound fetal measurements. 45 infants, thinner at birth, had experienced previous FGR (FGR+) regardless of birth weight. Growth pattern in the first four months of life was characterized by greater change in BMI z-score in FGR+ (+1.26+/−1.2 vs +0.58 +/−1.17 SD in FGR−) resulting in the restoration of BMI and of fat mass to values similar to FGR−, independently of caloric intakes. Growth velocity after 4 months was similar and BMI z-score and fat mass remained similar at 12 months of age. At both time-points, fetal growth velocity was an independent predictor of fat mass in FGR+. At one year, fasting insulin levels were not different but leptin was significantly higher in the FGR+ (4.43+/−1.41 vs 2.63+/−1 ng/ml in FGR−).

**Conclusion:**

Early catch-up growth is related to the fetal growth pattern itself, irrespective of birth weight, and is associated with higher insulin sensitivity and lower leptin levels after birth. Catch-up growth promotes the restoration of body size and fat stores without detrimental consequences at one year of age on body composition or metabolic profile. The higher leptin concentration at one year may reflect a positive energy balance in children who previously faced fetal growth restriction.

## Introduction

A robust regulatory physiologic system has evolved to maintain relative constancy of weight, an equilibrium broken by modern lifestyles leading to the development of obesity, type 2 diabetes and other metabolic disorders. Epidemiological studies have emphasized the role of changes in nutritional environment during fetal life or early infancy. The Dutch famine studies have clearly illustrated the relation between altered fetal growth induced by prenatal exposure to famine and increased risk of obesity and impaired glucose tolerance later in life [1], [2]. More recent studies have suggested that growth trajectory during early infancy, irrespective of birth weight, is important in determining later body size, fat mass and body composition [3], [4], [5]. In several birth cohorts, growth pattern during the first months of life is a predictor of obesity and metabolic risk, which effects are observed as early as in adolescence or childhood [6], [7], [8], [9].

Being born small for gestational age (SGA) is a clinical condition appropriate for the study of auxological and metabolic consequences of rapid postnatal growth. Indeed, most children born SGA show a rapid catch-up growth during the first year of life [10], [11]. In most cases but not all, this catch-up follows a phase of growth restriction during fetal development. This sequence represents a specific and relevant model to evaluate the auxological and metabolic consequences of early acceleration of postnatal growth. Some observations have emphasized that fetal growth restriction followed by rapid weight gain during early postnatal life may be a sequence promoting central adiposity, insulin resistance and ultimately type 2 diabetes and cardiovascular diseases [12], [13]. Fat mass excess and altered insulin sensitivity are suggested to be early events detectable at one year of age in children born SGA who experienced weight catch-up [14].

It has been reported that growth velocity at one month of age was correlated to fetal growth restriction [15]. However, how much of the catch-up growth relates to the fetal growth restriction itself during the first year of life and the exact window when catch-up growth becomes detrimental are not clearly identified. On the one hand, growth pattern during the three to four first months of life has been repeatedly reported to be associated with later clinical or biological markers of metabolic risk (dyslipidemia, increased blood pressure, abdominal obesity) in birth cohorts of young adults [16], [17].On the other hand, Barker *et al.* who first reported the association between birth weight and metabolic diseases, have recently reopened the debate by suggesting that high-risk subjects for cardiovascular diseases and diabetes would be the ones small at birth, thin at two years of age and who rapidly put on weight thereafter [18]. Before planning nutritional interventions it remains to be determined whether early catch-up growth is a phenomenon primarily dependent upon postnatal nutritional environment or whether it is mostly conditioned by the pattern of fetal growth. In other words, does this early acceleration of postnatal growth result from a conflict with postnatal nutrition or is it a compensatory phenomenon intended to replace infants on their own physiological growth curves?

The prospective evaluation of fetal growth is generally not included in the observation of birth cohorts precluding the study of the exact growth pattern and growth sequence that promote an increased risk of obesity and/or insulin resistance. In a cohort of newborns in whom fetal growth velocity had been recorded in a prospective and standardized manner and using the customized percentiles, a method that allows a precise evaluation of fetal growth restriction by identifying newborns who have failed to reach their genetic potential of growth [19], we have recently reported that fetal growth restriction by itself, independently from birth weight, is able to induce changes in body composition with a lower fat mass and changes in metabolism with a higher insulin sensitivity [20]. These changes reflect an adaptive process to the adverse fetal nutritional environment predisposing to rapid postnatal weight gain in a more favorable postnatal environment. Such changes were only seen in the newborns that previously experienced a profound fetal growth restriction but not in newborns with a regular or sub-regular fetal growth velocity. The same newborns may be later at high risk of developing of detrimental auxological and metabolic changes. The aim of the present study was therefore to relate the pattern of postnatal growth over the first year of life with fetal growth velocity and to study the simultaneous changes in body composition and metabolism.

## Methods

### Ethics statements

The Ethics Committee Ile-de-France 4 approved the study and written consent was obtained from both parents for all children.

### Study population and inclusion criteria

**Figure 1:**
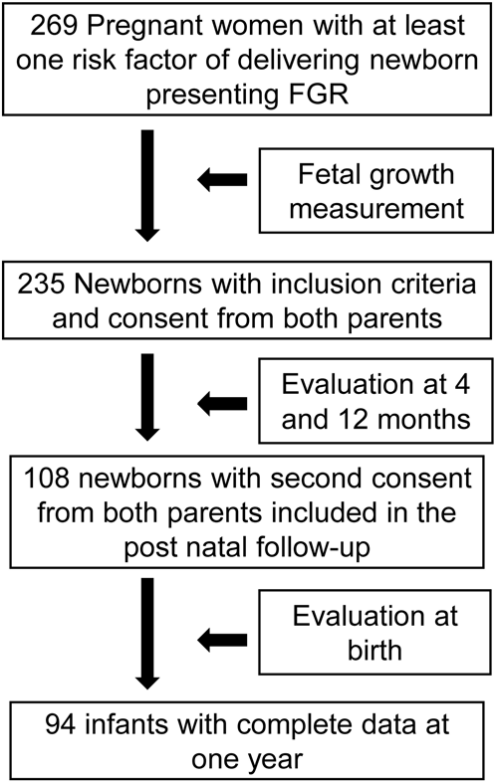
Study profile.

Results of auxological, anthropometric and metabolic assessments were obtained in a longitudinal study of 94 infants included at birth in the CASyMIR cohort (**figure 1**). The CASyMIR cohort included offspring born to Caucasian parents recruited during the first or second trimester of pregnancy in three French maternities (Robert Debré and Xavier Bichat Hospitals in Paris and Edouard Herriot Hospital in Lyon) and who were considered at risk of delivering small-for-gestational age babies. The inclusion criteria for the mother were the following: preexisting hypertension, smoking more than five cigarettes per day, a previous history of small for gestational age baby either in a previous pregnancy or among parents, a history of pregnancy-induced hypertensive disorder, maternal height less than 152 cm corresponding to -2SD of the mean height for French women, uterine malformations, abnormal uterine or umbilical artery Doppler and small fetal size at second trimester ultrasound examination (abdominal circumference and/or femoral length at 22 weeks of gestation). All newborns were evaluated at birth. Newborns presenting with fetal or congenital diseases that could affect fetal growth were excluded (TORCH infection, congenital malformation). Very low birth weight and gestational age below 36 weeks of gestation (WG) or newborns presenting with a severe neonatal condition were not included in the post-natal follow-up. Both parents have to give their consent for day 3 anthropometric evaluation; a second consent was given for the post-natal follow-up. 269 pregnant women were included in the study and 235 newborns were evaluated at birth. 127 parents accepted day 3 examination but not postnatal follow-up. The 108 remaining newborns that fulfilled inclusion criteria were evaluated at 4 and 9 months. Data for follow-up at one year were complete in 94 infants.

### Assessment of fetal growth

The date of conception was determined from the ultrasound examination at 12 weeks of gestation. Fetal growth was assessed every 4 weeks by ultrasound from 22 to 36 weeks of gestation by the same observer for each woman under a standardized protocol. Details of the procedure were given in previous publications [20], [21]. Estimated fetal weight (EFW) was calculated using the second Hadlock formula, which includes abdominal and head circumferences and femur length measurements [22]. The four measurements of EFW and birth weight were then converted to customized percentiles which were calculated for each case with a computer program which adjusts for parity, gender, maternal weight and height and ethnic group (Gestation related optimal weight (GROW) program, Software version 5.15. and Centile calculator software v5.12.1. March 2007, www.gestation.net) [19], [23]. Fetal growth velocity was calculated as the change in EFW percentiles from 22 WG until birth and was expressed as change in percentiles. We previously reported in the same cohort that a decrease by more than 25 percentiles in fetal growth was associated to metabolic and anthropometric changes in newborns. Consequently, the same cut-off limit was kept here to define fetal growth restriction and infants were divided in 2 groups regardless of birth weight: newborns with fetal growth restriction or FGR+ group and newborns with regular fetal growth or FGR− group.

### Post-natal follow-up

All children of the initial cohort born between June 2004 and February 2007 and who completed 1-year follow-up were included in the present study. Data concerning these infants were retieved at birth, at 4 and 12 months of age. No specific dietary recommendations were given to the parents and time of introduction of solid food (after 5 months of age) followed the French pediatric guidelines (http://www.sfpediatrie.com/fr/groupes-de-specialites/gfhgnp/comite-de-nutrition.html). During follow-up, infants who developed chronic diseases or who were taking medications that could interfere with growth or appetite were excluded.

### Measurements

A trained midwife or pediatrician performed the clinical measurements in all children at birth (in the maternities) and at 4 months and one year of age (at the Clinical Investigation Centre of Robert Debré Hospital in Paris and in the Neonatal care unit in Lyon). Weight was measured using an electronic scale to the nearest 10 grams. Supine length was measured to the nearest 0.5 cm with a standardized length board consisting of a fixed board for the infant's head and a movable board allowing feet to be placed perpendicular to the longitudinal axis of the infant. Skinfold thickness measurements were recorded at day 3 and at 4 and 12 months of age, on the left side of the body at four different sites (biceps, triceps, subscapular and suprailliacal), by the same trained pediatrician dedicated to the study. Two separate measurement were performed with a skinfold caliper (Harpenden skinfold caliper, Baty international, England) and the mean was recorded [24]. Upper arm circumference was measured at day 3 and 4 and 12 months of age. Two separate measurements were performed at mid arm and the mean recorded. Caloric intakes were recorded at 4 and 12 months of age using a questionnaire filled in by the parents with the pediatrician and providing detailed information on the intakes over the 3 days prior to each visit. If infants were breastfed at 4 months of age, frequencies and duration of mealtimes were recorded.

### Calculations

Body mass index (BMI = weight/lenght2) at birth, 4 and 12 months of age were calculated as appropriate weight for length indices as it was independent of length. Weight, length and BMI were converted into z-scores to adjust for age and sex using the French references curves [25], [26]. Changes in BMI z-score between birth and 4 months of age and between 4 and 12 months of age were calculated. Total subcutaneous fat mass was evaluated using the sum of the four skinfold thicknesses, and central subcutaneous fat mass using the central to total ratio: (supra-illiac skinfold+sub-scapular skinfold/sum of skinfold)*100 [24]. Percentage of body fat was derived from four skinfold mesurements from the equations of Brook and Siri [27], [28]. Lean body mass was evaluated using upper arm muscle area that derivates from skinfolds and upper arm circumference measurements. Upper arm muscle area (UMA) was obtained by subtracting total upper arm area (TUA) by upper arm fat area (UFA). TUA was calculated using the upper arm circumference (AC) with the following formula: TUA (cm^2^) = AC^2^/4 x π. UFA was calculated using the triceps skinfold with the following formula: UFA (cm^2^) = AC x (Triceps skinfold/2) [29]. At birth, small for gestational age (SGA) was defined as birth weight equal or below the 10^th^ and appropriate for gestational age (AGA) as birth weight above the 10^th^ percentile according to the French reference curves [30]. Insulin sensitivity was assessed using the index QUICKI (Quantitative insulin sensitivity check index) that was calculated as: QUICKI = 1/(log (fasting insulin)+log (fasting glucose)) [31].

### Assays

Hormonal analyses were performed at birth on a mixed venous and arterial cord blood sample. At 12 months of age, a fasting venous sample was obtained after an overnight fast. Glucose was measured immediately whereas samples for hormonal analysis were quickly centrifuged and serum was separated and stored at −80° until analysis. Serum insulin-like growth factor 1 (IGF-I) was measured by an immunoradiometric assay (IRMA) kit (IGF-I-RIACT) from Cis Bio international (Gif-sur-Yvette, France) and serum IGF-binding protein-3 (IGFBP-3) by an IRMA kit (ACTIVE IGFBP-3 IRMA) from DSL (Cergy Pontoise, France). Serum leptin was measured using a specific radioimmunoassay (Linco research, St Charles, USA). Sensitivity of the assay is 0.4 ng/ml. Intra-and inter- assay coefficients of variation are 5.2% and 8.7% respectively at 2.3 ng/ml. Serum insulin was measured by an IRMA kit (BI-INS-IRMA) from Cis Bio international ( Gif-sur-Yvette, France). Cross-reactivity with proinsulin and derived metabolites was less than 1%. Assay sensitivity was 3.0 pmol/L. Serum leptin concentrations were measured using a specific radioimmunoassay (Linco research, St Charles, USA). Sensitivity of the assay is 0.4 ng/ml. Intra- and inter- assay coefficients of variation are 5.2% and 8.7% respectively at 2.3 ng/ml.

### Statistical analyses

Comparisons of characteristics between groups were performed using the Chi-2 test for qualitative variables and the Student's t test for quantitative variables. Intragroups comparisons were performed using a non parametric test, the Wilkoxon rank-sum test. Relationships between continuous variables were assessed by simple linear regression analysis. We examined the effects of fetal growth velocity, post natal growth and birth weight on subcutaneous fat mass measured at 4 and 12 months of age, by using multivariate linear models. For each multivariate model, we reported the corresponding coefficient of determination, *R* ^2^ which expresses the proportion of variability in a data set that is accounted for by the statistical model. All models were statistically validated for assumptions of normality of residuals and absence of heteroscedasticity. Statistical analyses were performed using the SAS software version 9.1.3 for Windows (SAS statistical package, SAS institute, Meylan France).

## Results

### Anthropometric characteristics at birth, 4 months and 12 months of age

Characteristics of the infants at birth, 4 months and 12 months of age are given in table 1. Gender repartition was not different between the FGR+ and FGR− groups. In the first group, mean change in percentiles of EFW during fetal life was −52.76+/−19.6 percentiles over the second half of pregnancy. Unsurprisingly those FGR+ infants were lighter and thinner at birth as demonstrated by the lower BMI/birth weight z-scores and the lower sum of skinfolds at day 3 compared to FGR− infants. Lean mass was affected as well as indicated by the lower corrected UMA.

**Table 1:** Characteristics of infants at birth, 4 and 12 months of age according to fetal growth pattern.

	FGR−	FGR+
N =	49	45
*Change in percentiles*	*−6.2+/−11.6*	*−52.8+/−19.6* ¶
***Birth :***		
Gender (M/F)	18/31	23/22 ¶
Gestational age	38.9 +/−1.62	38.8+/−1.73 ¶
Birth Weight (g)	2913+/−566	2656+/−449
Birth Weigt (DS)	−0.76+/−1.15	−1.3+/−0.65 *
Length (cm)	47.9+/−2.72	47.16+/−2.41 *
Birth Lenght (DS)	−0.79+/1.18	−1.15+/−0.84
BMI (kg/m2)	12.64+/−1.24	12.06+/−1.02 *
BMI (DS)	−0.37+/−1.1	−0.91+/−0.81 *
Sum of skinfold (mm)	16.6+/−4.0	14.7+/−2.4*
Central to peripheral ratio	52.6+/−3.71	51.91+/−4.74
Upper arm fat mass area (cm^2^)	2.1+/−0.7	1.8+/−0.5 *
Upper arm muscular area (cm^2^)	5.88+/−1.5	5.25+/−1.1 *
***4 Months :***		
Weight (kg)	6.262+/−0.98	5.896+/−846
Weigt (DS)	−0.008+/−1.3	−0.48+/−1.1
Height (cm)	61.7+/−3.6	60.43+/−2.95
Height (DS)	−0,12+/−1.24	−0.58+/1.39
BMI (kg/m2)	16.4+/−1.56	16.08+/−1.4
BMI (DS)	0.19+/−1.1	0.14+/−1.03
***Δ IMC 0 to 4 months***	***0.58+/−1.17***	***1.26+/−1.2*** *
Breastfed n = (%)	11 (24.4)	6 (14.6)
Sum of skinfold (mm)	31.4+/−5.9	30+/−6.15
Central to peripheral ratio	48.41+/−5.04	48.79+/−5.8
Upper arm fat mass area (cm^2^)	6.33+/−1.8	6.17+/−1.6
Upper arm muscular area (cm^2^)	8.42+/−1.7	8.32+/−1.7
Fat Mass %	19.13+/−3.4	18.6+/−3.6
***12 Months :***		
Weight (kg)	9.271+/−1.3	9.147+/−1.03
Weigt (DS)	−0.26+/−1.3	−0.46+/−0.97
Height (cm)	74.11+/−2.6	73.6+/−2.5
Height (DS)	0.18+/−1.02	−0.14+/−1.0
BMI (kg/m2)	16.82+/−0.4	16.86+/−1.2
BMI (DS)	−0.4+/−1.24	−0.37+/−0.9
***Δ IMC 4 to 12 months***	***−0.63+/−0.24***	***−0.43+/−0.8***
Caloric intakes (kcal/kg)	92.78+/−18	92.95+/−23
Sum of skinfold (mm)	28.4+/−6.27	28.4+/−5.4
Central to peripheral ratio	46.21+/−5.71	47.6+/−5.13
Upper arm fat mass area (cm^2^)	6.79+/−1.9	6.67+/−1.7
Upper arm muscular area (cm^2^)	11.3+/−2.2	11.13+/−2.1
Fat Mass %	17.2+/−4.09	17.8+/−3.3

Data are given as mean +/− SD.

*p<0.05,

¶p<0.01

**Figure 2:**
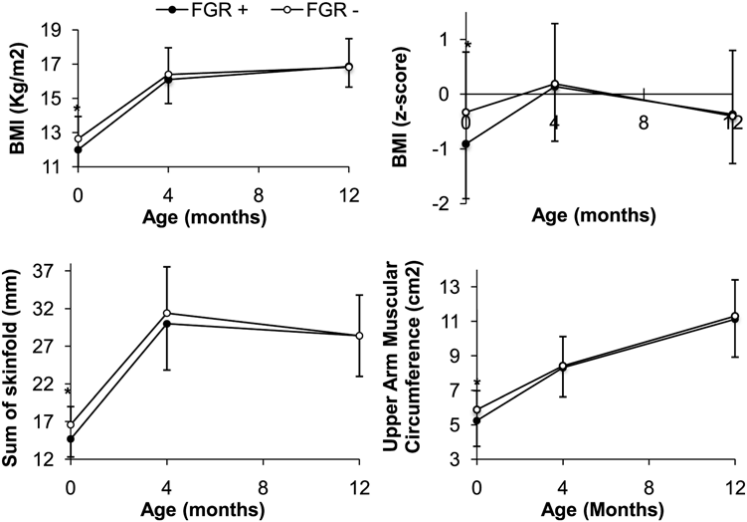
Anthropometric changes during and after catch-up growth. Changes in body mass index (A), body mass index z-score (B), skinfold thickness sum (C) and upper arm muscle area (D) between birth and one year of age in infants with (black circle) or without fetal growth restriction (white circle). *p<0.05

At 4 months, infants with FGR tended to remain lighter and shorter as shown by weight and height but there was no difference in BMI z-score in comparison to the FGR− infants. During the four first months of life, change in BMI z-score has been two-fold greater in the FGR+ group than in the FGR− group as shown in **figure 2A and 2B** **)**. The sum of skinfolds was no longer statistically different between the two groups indicating that catch-up growth was related to a restoration of fat mass. However, this restoration did not result in an excess of fat at 4 months of age since either sum of skinfold neither the percent of fat (as calculated by the Brooks equation) was not statistically different between the two groups **(** **figure 2C and 2D** **)**. Fat distribution was not affected by the accelerated growth as shown by the central to total ratio, which was not different between FGR+ and FGR− infants. Furthermore, catch-up growth was associated with fat-free mass restoration since UMA was no longer different between the two groups (figure 2D). The overall proportion of infants breastfed was low at this age and no difference was observed between the two groups. In the subgroups of non-breastfed infants, calorie intake was not different between FGR+ and FGR− (390+/−113 kcal/day vs 408+/−152 kcal/day p = 0.6 or 60.42+/−16.1 kcal/kg day vs 68.4+/−21.7 kcal/kg/day p = 0.12).

At 12 months of age, catch-up appears to be completed since weight, height and BMI (figure 2A and 2B) were similar between the two groups. At 12 months of age, again there were no differences either in total subcutaneous fat mass (sum of skinfolds, figure 2C) or in the calculated percent of fat mass or in the fat distribution (central to peripheral ratio). Calorie intake remained similar between the two groups. Between 4 and 12 months of age, growth velocity was similar in the two groups as reflected by the change in BMI z-score which was not significantly different (figure 1 and table 1). Calorie intake remained similar between the two groups.

**Figure 3:**
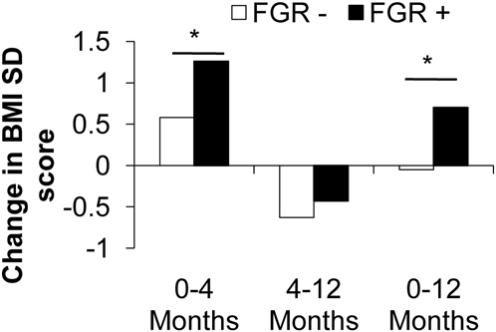
Change in BMI related to fetal growth pattern. Postnatal changes in body mass index between birth and 12 months of age in infants with (black bars) or without fetal growth restriction (white bars). *p<0.05

Infants in the FGR+ group only showed an important change in BMI z-score between birth and 12 months (+0.7+/−1.1 DS) whereas BMI z-score did not significantly change in the FGR− group (−0.05+/−1.1 DS) and this difference was found to be statistically significant (p<0.05) (**figure 3**).

### Determinants of sum of skinfold at 4 and 12 months of age

Table 2 and table 3 shows the determinants of sum of skinfolds, at 4 and 12 months respectively, in a multivariate analysis in the FGR+ and FGR− groups. In the FGR− group, the sum of skinfolds at four months, was positively related to birth weight and change in BMI z-score between birth and 4 months of age and this model explained 26% of the variance. In the FGR+ group, the sum of skinfolds at 4 months was also positively related to the same parameters but was negatively related to changes in EFW percentiles and these variables explained 52% of the variance in the data set. The sum of skinfolds at 12 months, was positively related to birth weight and to change in BMI z-score between birth and 4 months in FGR− infants. It was also related to change in BMI z-score between 4 and 12 months. 35% of the data set variance was in this case explained by these three parameters. Finally in the FGR+ group, the sum of skinfolds was negatively related to fetal growth only and not to the infants' birth weight. Fetal growth velocity, together with the early changes in BMI explained 41% of the variance of sum of skinfolds at 12 months in this group.

**Table 2:** Determinants of sum of skinfolds at 4 months of age in the two groups of infants.

	Dependent variable : Sum of skinfold at 4 months of age					
	*FGR−*			*FGR+*		
	β	95% CI	P value	β	95% CI	P value
Birthweight (g)	0.003	(0.012;0.001)	0.04	0.004	(−0.0002;0.009)	0.06
Change in percentiles of EFW between 22 and birth (percentiles)	0.05	(−0.12;0.22)	0.55	−0.12	(−0.22;−0.035)	0.008
Change in BMI 0–4 months (z- score)	2.79	(0.88;4.5)	0.004	3.28	(1.87;4.7)	<0.001

**Table 3:** Determinants of sum of skinfolds at 12 months of age in the two groups of infants.

	Dependent variable : Sum of skinfold at 12 months of age					
	*FGR−*			*FGR+*		
	β	95% CI	P value	β	95% CI	P value
Birthweight (g)	0.004	(−0.0007;0.008)	0.13	0.004	(−0.001;0.007)	0.1
Change in percentiles of EFW between 22 and birth (percentiles)	−0.02	(−0.17;−0.002)	0.78	−0.09	(−0.19; −0.015)	0.04
Change in BMI 0–4 months (z- score)	2,53	(0.61;3.59)	0.01	2.10	(0.61;4.43)	0.007
Change in BMI 4–12 months (z- score)	3.39	(0.92;5.86)	0.01	3.36	(0.8;5.97)	0.008

### Hormonal changes between birth and 12 months of age

Hormonal data at birth and at 12 months of age are given in table 4. As previously reported, cord blood Insulin, IGF-1 and IGFBP3 were lower at birth, in the FGR+ group. At 12 months, they were no longer different. As previously reported, QUICKI at birth tended to be higher in the FGR+ group whereas it was similar in the two groups at 12 months. At birth, cord blood leptin was lower in the FGR group. Interestingly the venous concentration was higher at 12 months despite the similar level of fat mass in the 2 groups. In both group, fat mass and BMI z-score were correlated with leptin concentration (data not shown). In multivariate analysis, leptin at 12 months was negatively related to fetal growth velocity (β = −0.004, p = 0.05) and positively related to change in BMI z-score between birth and 4 months (β = 0.12, p = 0.008).

**Table 4:** Metabolic characteristics of at birth, 4 and 12 months of age according to fetal growth pattern.

	FGR−	FGR+
***Birth***		
Glycemia (mmol/l)	3.97+/−1.2	4.57+/−1.3
Cord Insulin (mUI/l)	4.64+/−2.1	2.0+/−2.6 ¶
Cord IGF-1 (ng/ml)	73.62+/−35.2	47.26+/−36.4 ¶
Cord IGF-BP3 (µg/ml)	1.25+/−0.53	0.8+/−0.39 *
Cord Leptin (ng/ml)	8.34+/−2.69	4.02+/−3.0 ¶
QUICKI	0.19+/−0.03	0.22+/−0.03 *
***12 Months***		
Glycemia (mmol/l)	4.6+/−0.4	4.66+/−0.5
Insulin (mUI/l)	2.28+/−1.96	2.38+/−2.2
IGF-1 (ng/ml)	82.8+/−28.9	80.0+/−27.9
IGF-BP3 (µg/ml)	3.09+/−0.55	3.15+/−0.1
Leptin (ng/ml)	2.63+/−1.00	4.43+/−1.41 *
QUICKI	0.23+/−0.09	0.23+/−0.08

Data are given as mean +/− SD.

*p<0.05,

¶p<0.01

## Discussion

We report here infants growth pattern of weight, lean and fat mass in the first year of life in relation with fetal growth pattern but regardless of birth weight. It is commonly thought that catch-up growth is a phenomenon only seen in infants born small for gestational age and a consequence of small birth measurements [32]. Our data emphasize that catch-up growth is more likely linked to fetal growth restriction *per se* than to birth weight, representing an adaptive and compensatory phenomenon of rather short duration (first months of life and less than one year) to compensate for the previous FGR due to unfavorable fetal environment. Indeed, in our population, fetal growth restriction is followed by an accelerated growth in order to restore body size, fat stores and body composition that were altered during fetal life. This accelerated growth took place early in life and is of short duration as the auxological differences between the two groups of newborns were no longer present at 4 months of age. Accordingly, the physiological function of this “fetal life-induced” accelerated growth seemed to be the correction of body size as it halted when BMI was restored and growth velocity returned to similar values as the ones seen in infants with no FGR. However this phenomenon does not develop here in the detriment of body composition at one year of age. Accelerated growth is associated with accelerated gain in both fat and fat-free mass in the first months of life leading to similar values in both compartments at four months of age in the two groups.

The fetal programming concept points to the possible deleterious role played by catch-up growth in body composition and in metabolic complications. Our results rather highlight that “catch-up” growth during the first year of life is a physiological and compensatory process that followed a period of growth deficiency induced by an altered fetal environment and which aims are to replace the organism on the “physiological” growth trajectory and to restore body composition. Indeed, in our study population, subcutaneous fat mass which was decreased at birth in the FGR+ group was not only linked to the increase in BMI at both 4 and 12 months of age - as expected- but more interestingly was negatively linked to fetal growth velocity. The more important the fetal growth restriction and the lower the fat mass at birth, the greater the fat mass was in the first year of life. However, this accelerated growth of the adipose tissue does not induce excessive fat mass or alteration in its distribution at the age of one year. Furthermore, the process appears limited in time and there does not seem to be a prolonged imprinting on fat growth in the first year of life. When fat stores are restored and growth velocity returns to normal values, fat accretion becomes similar in both groups.. Likewise, catch-up growth does not induce detectable insulin resistance at the end of the first year of life.

Interestingly, the accelerated growth velocity during catch-up appears here as an intrinsic phenomenon. First, although precise data on the caloric intakes are lacking for the first weeks of life, we didn't find accelerated growth and fat deposition to be associated with hyperphagia at four months of age, since calorie intakes measured in the bottle-fed infants are similar whether previous FGR or not. Second, growth velocity returned to physiological values when body composition was restored. Third, the increased leptin concentration could favor a positive energy balance promoting this catch-up growth.

The short follow-up of our study and the small number of children do not allows us to give definitive conclusions on the link between catch-up growth and later metabolic disease. However, fetal growth restriction followed by rapid postnatal weight gain has been clearly pointed as a risk factor of deleterious changes in insulin sensitivity and body composition. Our data could prompt to reconsider the timing of such changes and are actually not in agreement with previous data indicating the early deleterious effect of catch-up growth. It is important to emphasize that catch-up growth is not always similar to weight gain. Catch-up growth implies a weight gain appropriate for height gain. Ong *et al* first defined catch-up growth as a gain of weight by more than 0.67 DS in the first years of life and reported the deleterious consequences on the later risk of obesity [6]. In the study by Soto *et al* catch-up growth in SGA infants was similarly defined and was indeed associated with increased fasting insulin at one year of age in SGA infants [14]. However, in the subgroup of SGA infants with such a catch-up in weight, weight gain was two-fold greater than the one in height, between birth and one year of age. This greater weight rather than height gain may suggest that insulin resistance was not due to a “physiological” catch-up growth but to the excessive gain in weight and fat. By comparison, weight gain in our cohort was similar in z-score to height gain (respectively 0.84 and 1.01 DS). In the study by Veening *et al* in SGA children, catch-up growth was defined by height gain from birth to age 7 [33]. Decreased insulin sensitivity was found only in SGA children that experienced catch-up with excessive current BMI. Such a distinction between “developmental” catch-up and excessive weight gain could furthermore explain some discordant results on the relationship between birth measurements and the metabolic risk. A recent study reported the determinants of insulin sensitivity in young adults born either AGA or SGA [34]. The only independent determinant of insulin sensitivity was fat gain from birth rather than birth size itself or catch-up growth (the definition of which was based on height). Only one group has followed anthropometric and metabolic changes in SGA and AGA children from 2 to 6 years of age [13], [35]. At inclusion, catch-up growth was completed in SGA children and BMI or percent fat were similar to AGA children. Yet, fat gain was greater in SGA children between 2 and 6 years of age and was associated with changes in insulin sensitivity. If BMI was similar in both groups at 4 years of age, it was greater in SGA children at 6. These results indicate that after the period of early catch-up, growth infants enter a second critical phase of auxological changes, from 2 years onward, where weight gain and changes in BMI would be associated to negative changes in fat mass and in insulin sensitivity.

With consideration to these previous studies, our results prompt us to revise the natural history of fetal programming and insulin resistance. Hence, a deleterious fetal environment and more specifically fetal growth restriction induces adaptive changes in the fetal metabolism in order to adjust to a predicted poor postnatal environment. Therefore, these changes ensure not only immediate fetal survival but also postnatal survival. Fetal growth restriction compromises growth of nutrient storage organs such as adipose tissue and this is compensated by adaptive metabolic changes such as greater insulin sensitivity to favour energy efficiency and utilization of alternate source of substrates. These changes would result to a thin newborn whose metabolism would be programmed to storage and efficient energy utilization. Indeed, metabolic changes, as suggested by the developmental mismatch theory [36], are in “conflict” with an adequate postnatal environment. This “conflict” ultimately results to the well-described catch-up growth. Further, our results highlight that such catch-up growth does not strictly always overlap with excessive postnatal weight gain especially when it follows a fetal growth restriction. In this particular situation, catch-up growth seems to be an endogenous and physiological process dedicated to the restoration of body size and composition without negative consequences on insulin metabolism. Later in childhood, the higher leptin secretion observed at one year of age could play an important role in the future metabolic and auxological deleterious changes.

Leptin levels has been widely accepted as a marker of fat mass, and leptin receptors are expressed in several tissues including fetal cartilage, bone lung kidney or hypothalamus suggesting that leptin may exert its biological functions in the fetus and/or early life. Cord leptin has been recently reported to be a predictor of weight gain over the first six months of age and of anthropometric outcomes at 3 years of life [37]. In healthy children, the role of leptin is first to regulate energy homeostasis by modulating energy intake and expenditure. It also regulates several nueroendocrine axes including the growth hormone-IGF axis [38], [39]. The lower neonatal concentration of leptin, already reported in SGA newborns [40], [41], [42] could impair the development of hypothalamic pathways. Recent studies in rodents have illustrated the plasticity of arcuate nucleus projections in the neonatal period and have described a role for leptin as being a neurotrophic signal for the development of hypothalamic circuits [43], [44], [45]. Finally, although no clear effects on calorie intakes was evidenced during the first year of life, the reduced neonatal concentration of leptin may have long-term consequences on food intake, nutrient storage and body weight control. On the other hand, the higher leptin concentration observed here as early as one year of age and consistently reported in SGA individuals [40], [46] could contribute to a positive energy balance and later fat mass accretion, which in turn will induce insulin resistance leading to a vicious cycle of escalating metabolic diseases. The short duration of our follow-up is clearly a restriction to such a conclusion; however, results from previous human or animal studies are concordant with this idea. A recent work on an animal model of nutritional induced fetal growth restriction has pointed the crucial role played by leptin signalling modulation in the puppets. In this compromised infant rats, leptin administration shifted the developmental programme from a metabolically unhealthy to a healthy one [47].

In conclusion, early catch-up growth following fetal growth restriction promotes restoration of fat stores and does not induce unfavourable changes either in body composition or in insulin sensitivity at one year of age. However, changes in leptin secretion and/or sensitivity occurring in the first year of life could be an important metabolic marker of fetal programming. Perturbations in perinatal nutrition that alter leptin sensivity could have lifelong consequences for control of body weight, fat mass and insulin sensitivity, but the real relevance of our observation remains to be further demonstrated. Interventions in early life in order to promote weight gain by increasing caloric or protein intake have shown that modulation of infant growth during first months of life could lead to deleterious changes in blood pressure, altered flow-mediated endothelial dilatation [48], [49], [50]. Our results support that interventions in the first months of life may not be appropriate with respect to the metabolic risk during such a window of plastic development of numerous organs.
